# Supplementary material for: The potassium channel KCNJ13 is essential for smooth muscle cytoskeletal organization during mouse tracheal tubulogenesis
Source: Nat Commun. 2018 Jul 19;9:2815. doi: 10.1038/s41467-018-05043-5 (PMC6052067; doi:10.1038/s41467-018-05043-5)
Supplement: Supplementary file 1 — Supplementary Information [file 41467_2018_5043_MOESM1_ESM.pdf]

Supplementary Information

The potassium channel KCNJ13 is essential for smooth muscle cytoskeletal organization during mouse tracheal tubulogenesis

Yin et al.

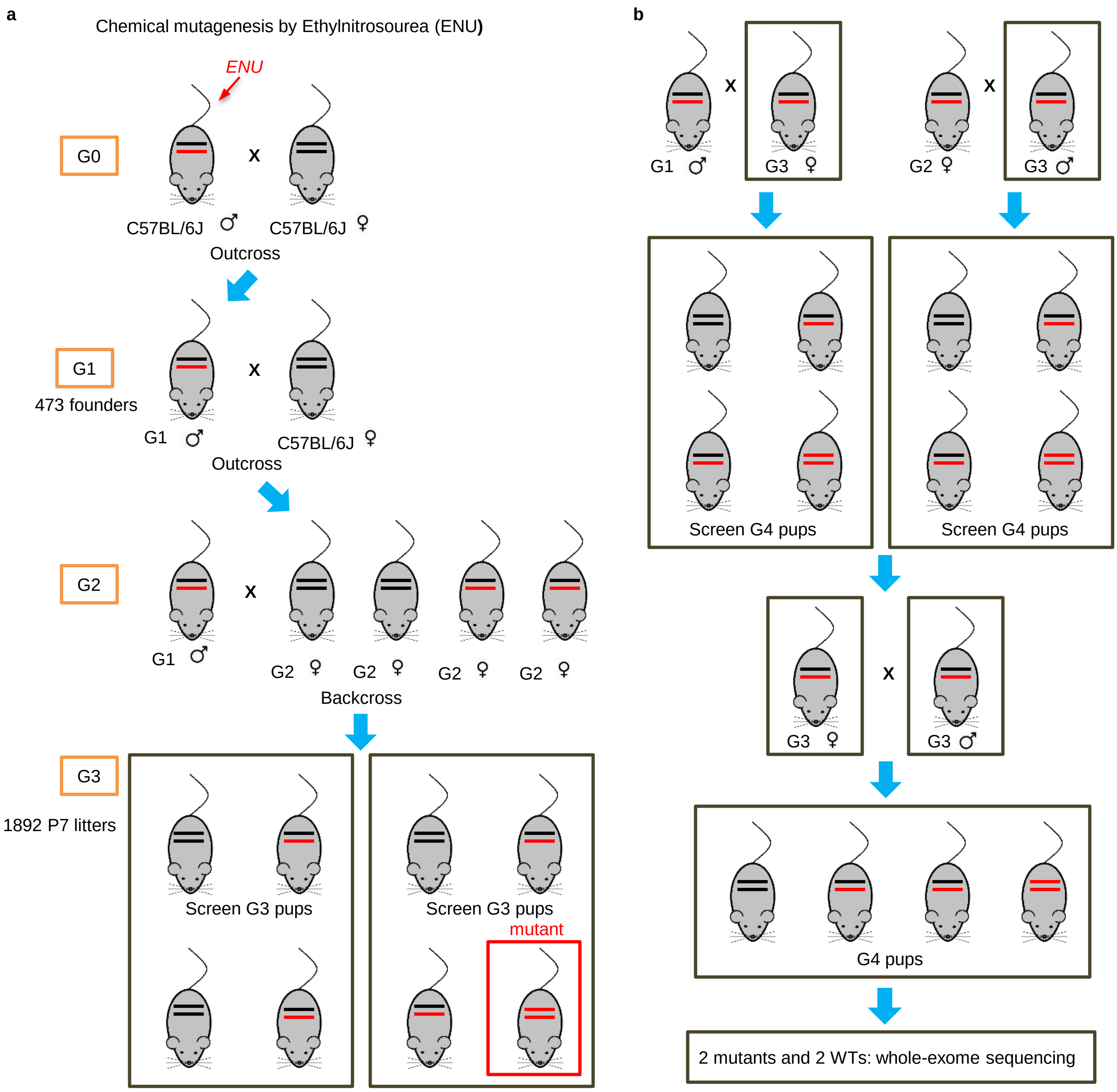

Supplementary Figure 1. Workflow for mouse forward genetic screen and mutation recovery. (**a**) Backcross breeding scheme carried out to generate G3 homozygous mutants with all progeny from one G1 male founder. (**b**) Pipeline for mutant recovery and mutation identification.

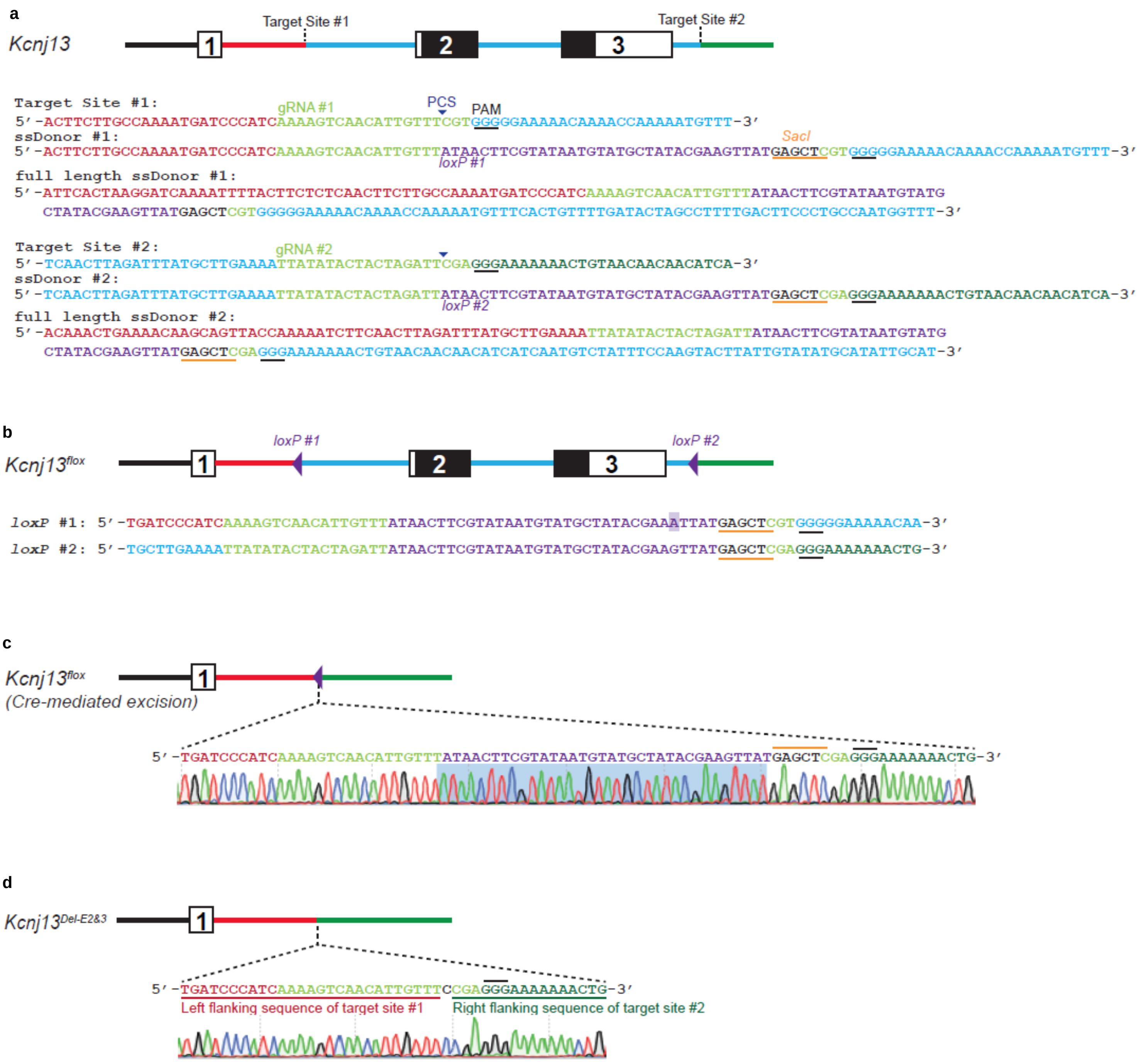

Supplementary Figure 2. Generation of mouse *Kcnj13* floxed and deletion alleles. (**a**) The CRISPR/Cas9 system was used to generate mice carrying *loxP* sites flanking exons 2 and 3 of *Kcnj13*. gRNA #1 and gRNA #2 were used to target 185 bp single stranded donor sequences (ssDonor #1 and #2) carrying *loxP* sites to intronic sites flanking exons 2 and 3. *SacI* sites were included in the ssDonor sequences to verify PCR product identity. (**b**) One mouse carrying two *loxP* sites in *cis* (*Kcnj13*^{flox}) was recovered, and sequences across the two *loxP* sites following germline transmission of the allele are shown. Although *loxP* #1 has a single base pair mutation (A, shaded), it does not appear to affect Cre-mediated excision substantially. (**c**) Cre expression in mice carrying the floxed *Kcnj13* allele results in a 9 kb deletion with a single residual *loxP* site remaining. (**d**) In addition to the floxed allele, mice with a deletion of exons 2 and 3 of *Kcnj13* were also recovered. This *Kcnj13* deletion allele (*Kcnj13*$^{Del\text{-}E2\&3}$) contains a nearly

perfect 9 kb deletion between the two predicted cleavage sites, with the exception of a single additional base pair (a "C" shown in black between the right and left flanking sequences). DNA sequence colors correspond to specific labeled features. Putative Cleavage Site (PCS).

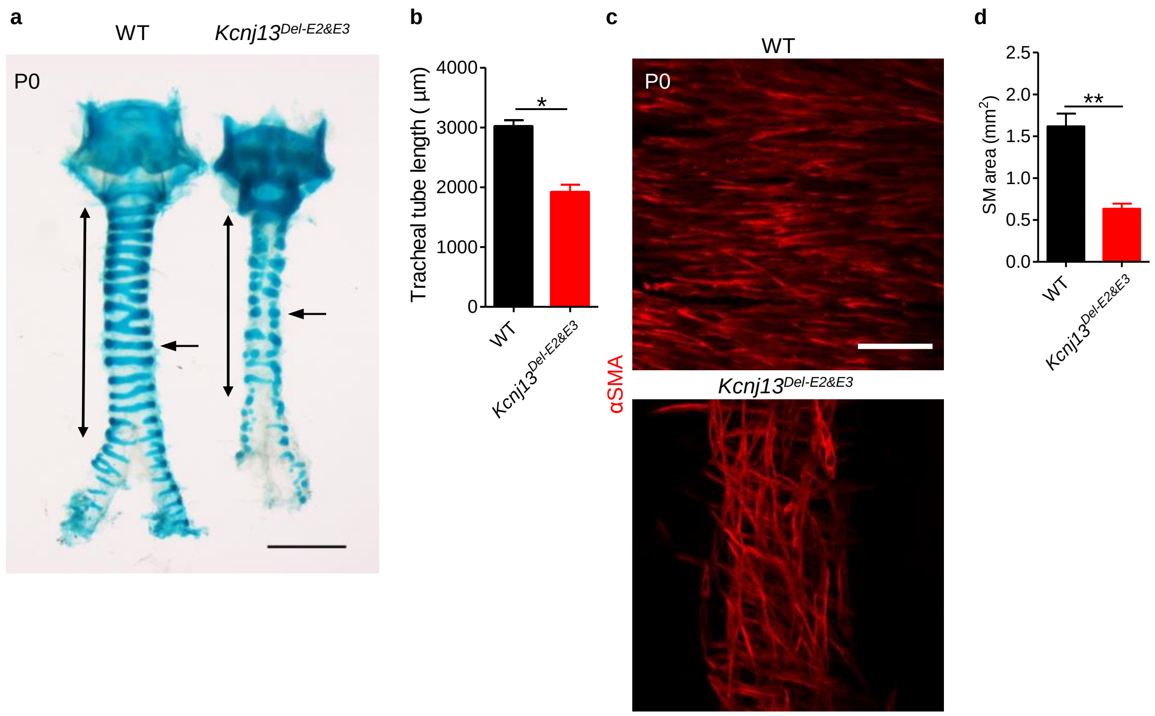

Supplementary Figure 3. ***Kcnj13$^{Del\text{-}E2\&E3}$*** **mice exhibit tracheal tube formation defects.** (**a**) Representative image of ventral view of wholemount tracheas stained with alcian blue from P0 WT (n=12) and *Kcnj13$^{Del\text{-}E2\&E3}$* mice (n=12). Double-sided arrows indicate tracheal tube length. Arrows point to tracheal cartilage rings. (**b**) Quantification of P0 WT (12) and *Kcnj13$^{Del\text{-}E2\&E3}$* (12) tracheal tube length. (**c**) Representative images of dorsal views of wholemount tracheas stained for αSMA (red) from P0 WT (n=8) and *Kcnj13$^{Del\text{-}E2\&E3}$* mice (n=8). (**d**) Quantification of P0 WT (n=8) and *Kcnj13$^{Del\text{-}E2\&E3}$* (n=8) SM area. Scale bars: 1000 µm (**a**), 50 µm (**c**). *$P < 0.05$; **$P < 0.01$; Unpaired Student's *t*-test, mean ± s.d.

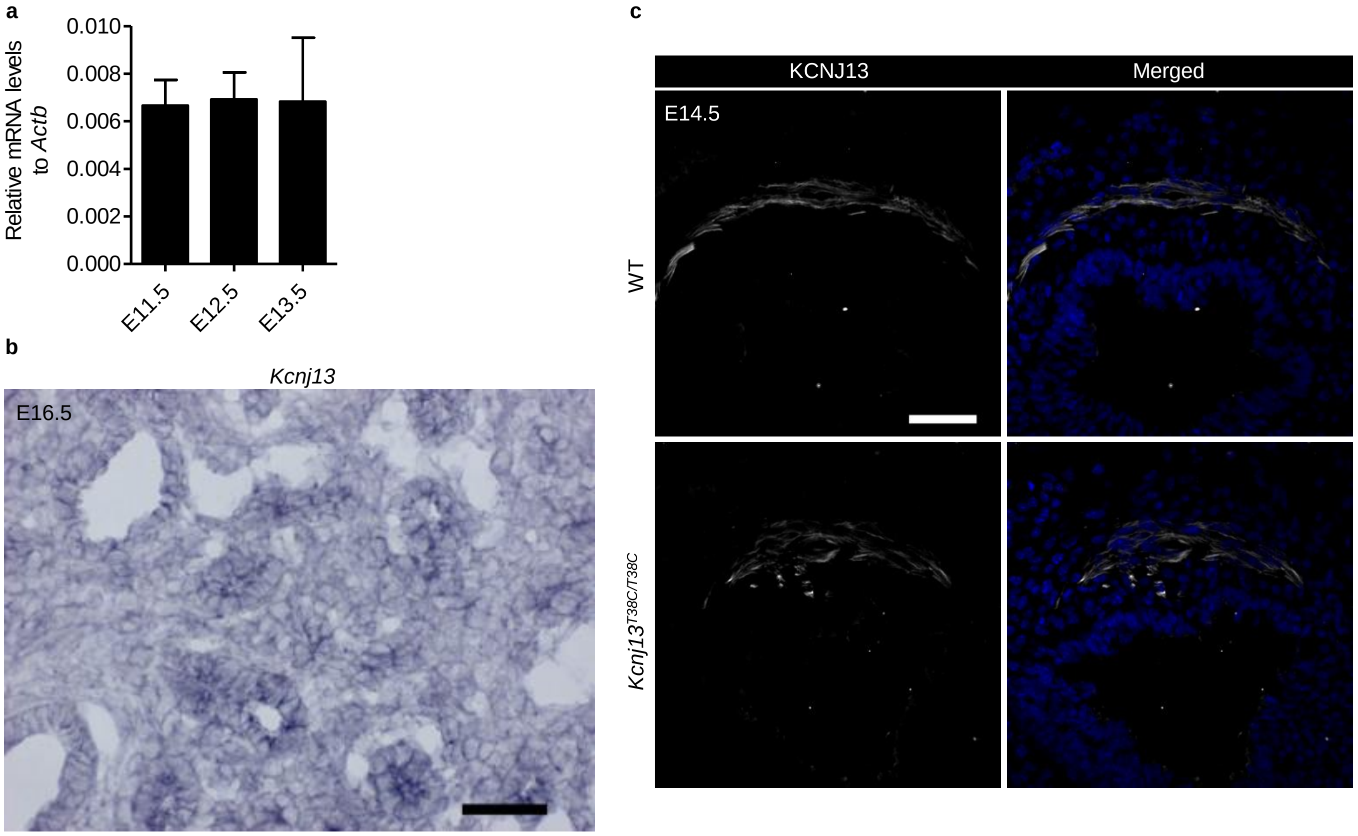

Supplementary Figure 4. *Kcnj13* mRNA expression and KCNJ13^{L13P} expression in tracheal SM cells. (**a**) RT–qPCR analysis of *Kcnj13* expression in WT tracheas (n=5 per stage). (**b**) mRNA in situ hybridization for *Kcnj13* in E16.5 WT lung tissue sections (n=5). (**c**) Immunostaining for KCNJ13 (red) and DAPI staining (blue) of transverse sections of E14.5 WT (n=8) and *Kcnj13$^{T38C/T38C}$* tracheas (n=8). Scale bars: 50 µm.

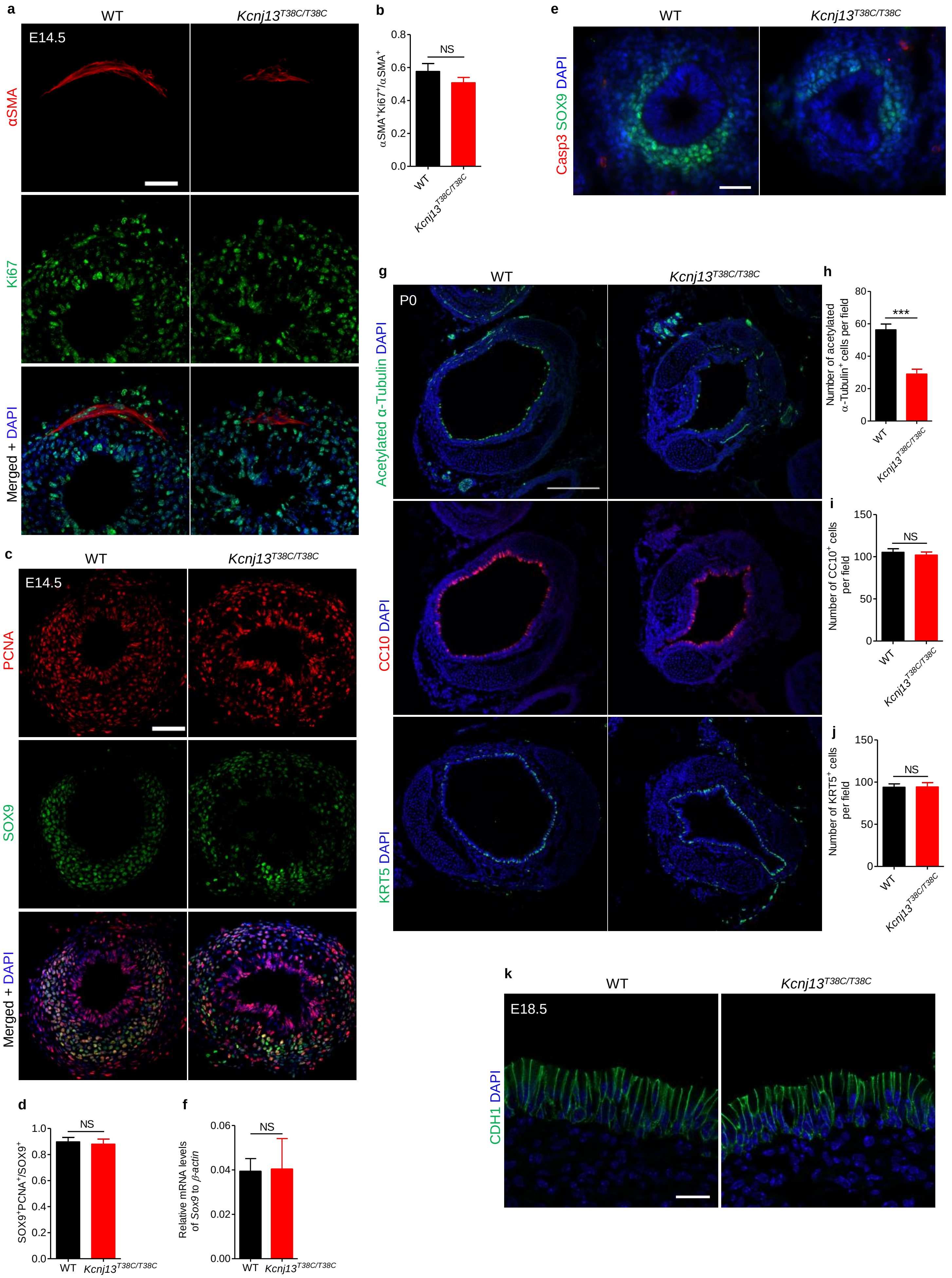
a
WT
Kcnj13T38C/T38C
E14.5
αSMA
Ki67
Merged + DAPI
b
αSMA+Ki67+/αSMA+
NS
0.0
0.2
0.4
0.6
0.8
WT
Kcnj13T38C/T38C
c
E14.5
PCNA
SOX9
d
SOX9+PCNA+/SOX9+
1.0
e
Casp3 SOX9 DAPI
f
Relative mRNA levels of Sox9 to β-actin
0.00
0.02
0.04
0.06
g
P0
Acetylated α-Tubulin DAPI
CC10 DAPI
KRT5 DAPI
h
Number of acetylated α-Tubulin+ cells per field
***
0
20
40
60
80
i
Number of CC10+ cells per field
50
100
150
j
Number of KRT5+ cells per field
k
E18.5
CDH1 DAPI

Supplementary Figure 5. *Kcnj13*$^{T38C/T38C}$ mice exhibit WT-like proliferation and apoptosis rate of SM cells and SOX9+ mesenchymal cells but reduced multiciliated cell differentiation. (**a**) Immunostaining for αSMA (red) and Ki67 (green) and DAPI staining (blue) of transverse sections of E14.5 WT (n=7) and *Kcnj13*$^{T38C/T38C}$ (n=7) tracheas. (**b**) Percentage of αSMA^{+} cells that are Ki67^{+}. (**c**) Immunostaining for PCNA (red) and SOX9 (green) and DAPI staining (blue) of transverse sections of E14.5 WT (n=7) and *Kcnj13*$^{T38C/T38C}$ (n=7) tracheas. (**d**) Percentage of SOX9+ cells that are PCNA+. (**e**) Immunostaining for cleaved caspase-3 (Casp3, red) and SOX9 (green) and DAPI staining (blue) of transverse sections of E13.5 WT (n=6) and *Kcnj13*$^{T38C/T38C}$ (n=6) tracheas. (**f**) RT-qPCR analysis of *Sox9* expression in E14.5 WT (n=6) and *Kcnj13*$^{T38C/T38C}$ (*n*=6) tracheas. (**g**) Immunostaining for acetylated alpha-tubulin (multiciliated cells; green), CC10 (club cells; red) and KRT5 (basal cells; green) and DAPI staining (blue) of transverse sections of P0 WT (n=7) and *Kcnj13*$^{T38C/T38C}$ (n=7) tracheas. (**h**) Quantification of acetylated alpha-tubulin^{+} cells in each section of P0 WT (n=7) and *Kcnj13*$^{T38C/T38C}$ (n=7) tracheas. (**i**) Quantification of CC10^{+} cells in each section of P0 WT (n=7) and *Kcnj13*$^{T38C/T38C}$ (n=7) tracheas. (**j**) Quantification of KRT5^{+} cells in each section of P0 WT (n=7) and *Kcnj13*$^{T38C/T38C}$ tracheas (n=7). (**k**) Immunostaining for CDH1 (epithelial cells; green) and DAPI staining (blue) of transverse sections of E18.5 WT (n=7) and *Kcnj13*$^{T38C/T38C}$ (n=7) tracheas. Scale bars: 200 μm (**g**), 50 μm (**a, c**), 20 μm (**e**, **k**). ***$P < 0.001$; NS, not significant; Unpaired Student's *t*-test, mean ± s.d.

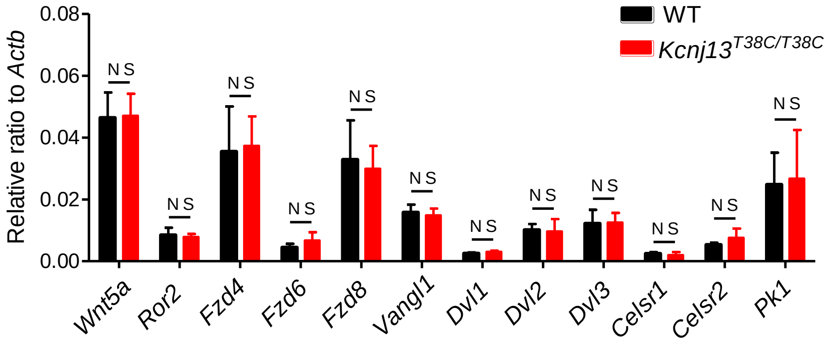

Supplementary Figure 6. ***Kcnj13**$^{T38C/T38C}$* **tracheas exhibit WT-like expression levels of Wnt/PCP pathway genes.** RT–qPCR analysis of *Wnt5a*, *Ror2*, *Fzd4*, *Fzd6*, *Fzd8*, *Vangl1*, *Dvl1*, *Dvl2*, *Dvl3*, *Celsr1*, *Celsr2* and *Pk1* expression in E14.5 WT (n=5) and *Kcnj13*$^{T38C/T38C}$ (*n*=5) tracheas. NS, not significant; Unpaired Student's *t*-test, mean ± s.d.

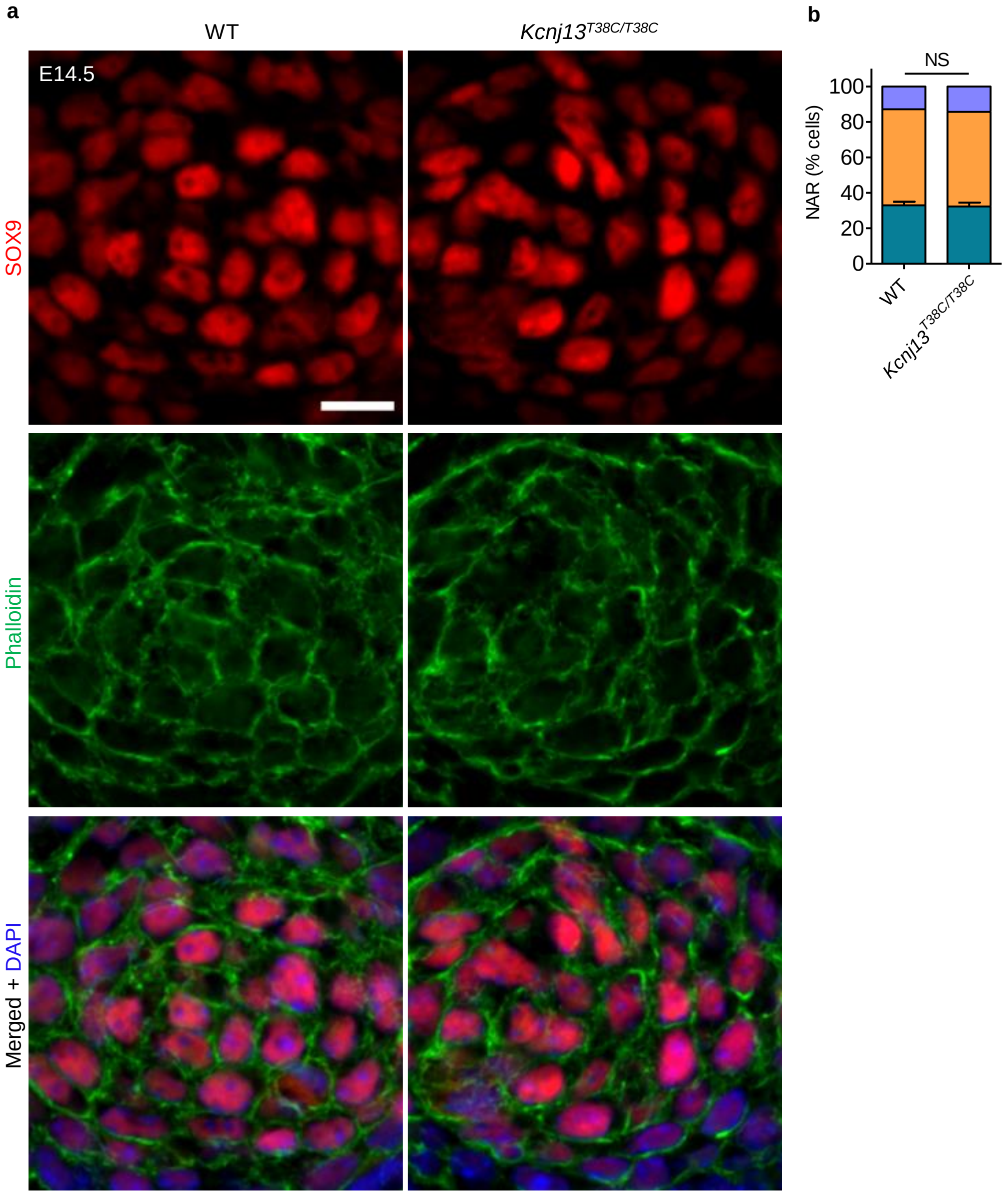

Supplementary Figure 7. ***Kcnj13$^{T38C/T38C}$*** **tracheas exhibit WT-like SOX9+ mesenchymal cell shape.** (**a**) Immunostaining for SOX9 (red) and phalloidin (green) and DAPI (blue) staining of transverse sections of E14.5 WT (n=7) and *Kcnj13$^{T38C/T38C}$* (n=7) tracheas. (**b**) Quantification of cell nuclear aspect ratio (NAR) of E14.5 WT (n=7) and *Kcnj13$^{T38C/T38C}$* (n=7) SOX9+ mesenchymal cells. Scale bar: 10 μm (**a**). NS, not significant; Unpaired Student's *t*-test, mean ± s.d.

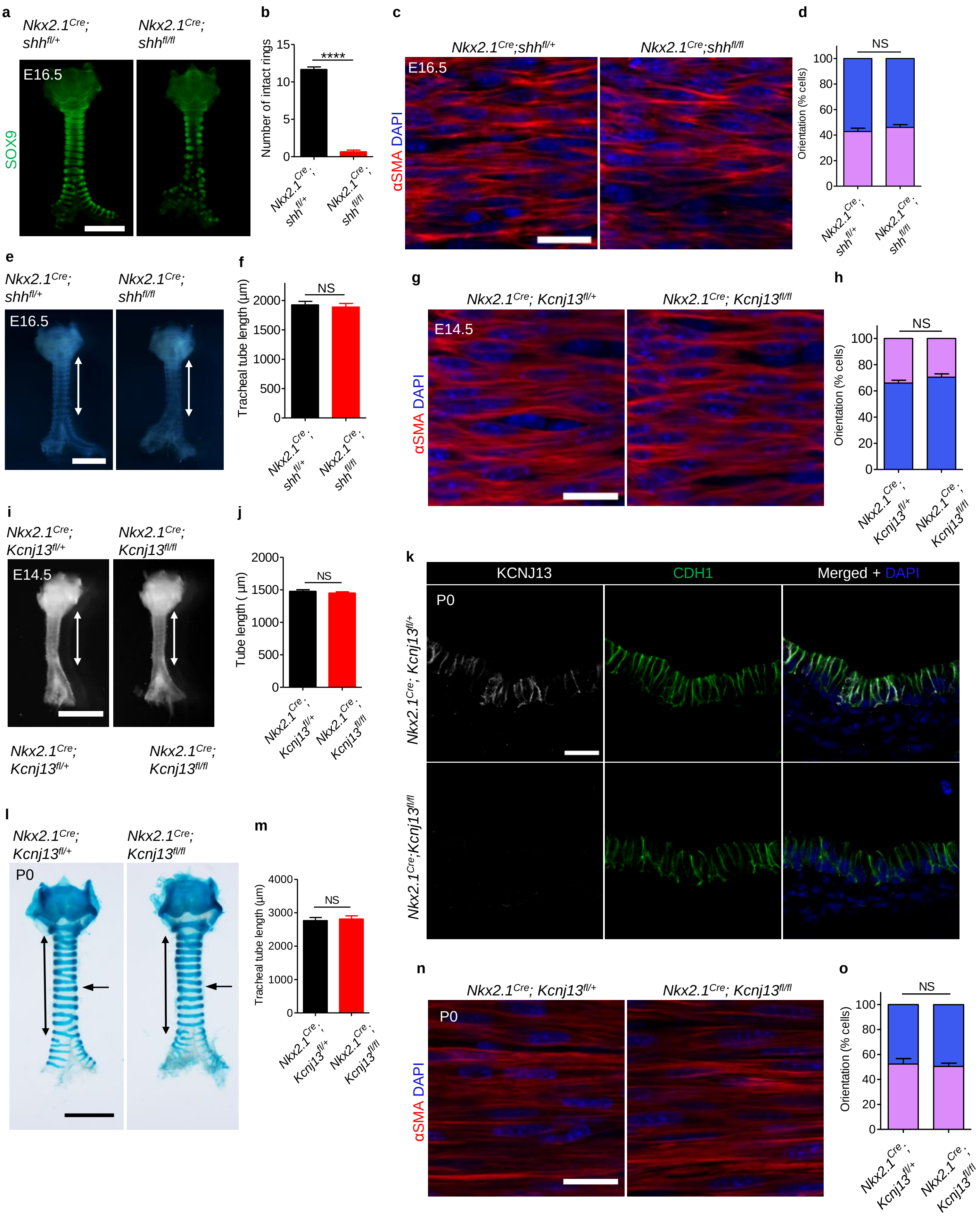

Supplementary Figure 8. ***Nkx2.1**^{Cre}**;shh**$^{flox/flox}$* **and** ***Nkx2.1**^{Cre}**;Kcnj13**$^{flox/flox}$* **mice exhibit WT-like SM cell alignment and tracheal tube elongation.** (**a**) Immunostaining for SOX9 (green) in ventral views of E16.5 control (n=7) and *Nkx2.1*^{Cre}*;shh*$^{flox/flox}$ (n=7) tracheas. (**b**) Quantification of intact tracheal cartilage rings in

E16.5 control (n=7) and *Nkx2.1^{Cre};shh$^{flox/flox}$* (n=7) tracheas. (**c**) Immunostaining for αSMA (red) and DAPI staining (blue) in dorsal views of E16.5 control (n=7) and *Nkx2.1^{Cre};shh$^{flox/flox}$* (n=7) tracheas. (**d**) Quantification of E16.5 control (n=7) and *Nkx2.1^{Cre};shh$^{flox/flox}$* (n=7) tracheal SM cell orientation. (**e**) Representative images of E16.5 control (n=7) and *Nkx2.1^{Cre};shh$^{flox/flox}$* (n=7) tracheas. Double-sided arrows indicate tracheal tube length. (**f**) Quantification of E16.5 control (n=7) and *Nkx2.1^{Cre};shh$^{flox/flox}$* (n=7) tracheal tube length. (**g**) Immunostaining for αSMA (red) and DAPI staining (blue) in dorsal views of E14.5 control (n=6) and *Nkx2.1^{Cre};Kcnj13$^{flox/flox}$* (n=6) tracheas. (**h**) Quantification of E14.5 control (n=6) and *Nkx2.1^{Cre};shh$^{flox/flox}$* (n=6) tracheal SM cell orientation. (**i**) Representative images of E14.5 control (n=6) and *Nkx2.1^{Cre};Kcnj13$^{flox/flox}$* (n=6) tracheas. Double-sided arrows indicate tracheal tube length. (**j**) Quantification of E14.5 control (n=6) and *Nkx2.1^{Cre};Kcnj13$^{flox/flox}$* (n=6) tracheal tube length. (**k**) Immunostaining for KCNJ13 (red) and CDH1 (green) and DAPI staining (blue) of transverse sections of P0 control (n=5) and *Nkx2.1^{Cre};Kcnj13$^{flox/flox}$* (n=5) tracheas. (**l**) Representative images of ventral views of wholemount tracheas stained with alcian blue from P0 control (n=5) and *Nkx2.1^{Cre};Kcnj13$^{flox/flox}$* mice (n=5). Double-sided arrows indicate tracheal tube length. Arrows point to tracheal cartilage rings. (**m**) Quantification of P0 control (n=5) and *Nkx2.1^{Cre};Kcnj13$^{flox/flox}$* (n=5) tracheal tube length. (**n**) Immunostaining for αSMA (red) and DAPI staining (blue) in dorsal views of P0 control (n=6) and *Nkx2.1^{Cre};Kcnj13$^{flox/flox}$* (n=6) tracheas. (**o**) Quantification of P0 control (n=6) and *Nkx2.1^{Cre};shh$^{flox/flox}$* (n=6) tracheal SM cell orientation. Scale bars: 1000 μm (**a, e, i, o**), 20 μm (**c, g, k, n**). ****$P < 0.0001$; NS, not significant; Unpaired Student's *t*-test, mean ± s.d.

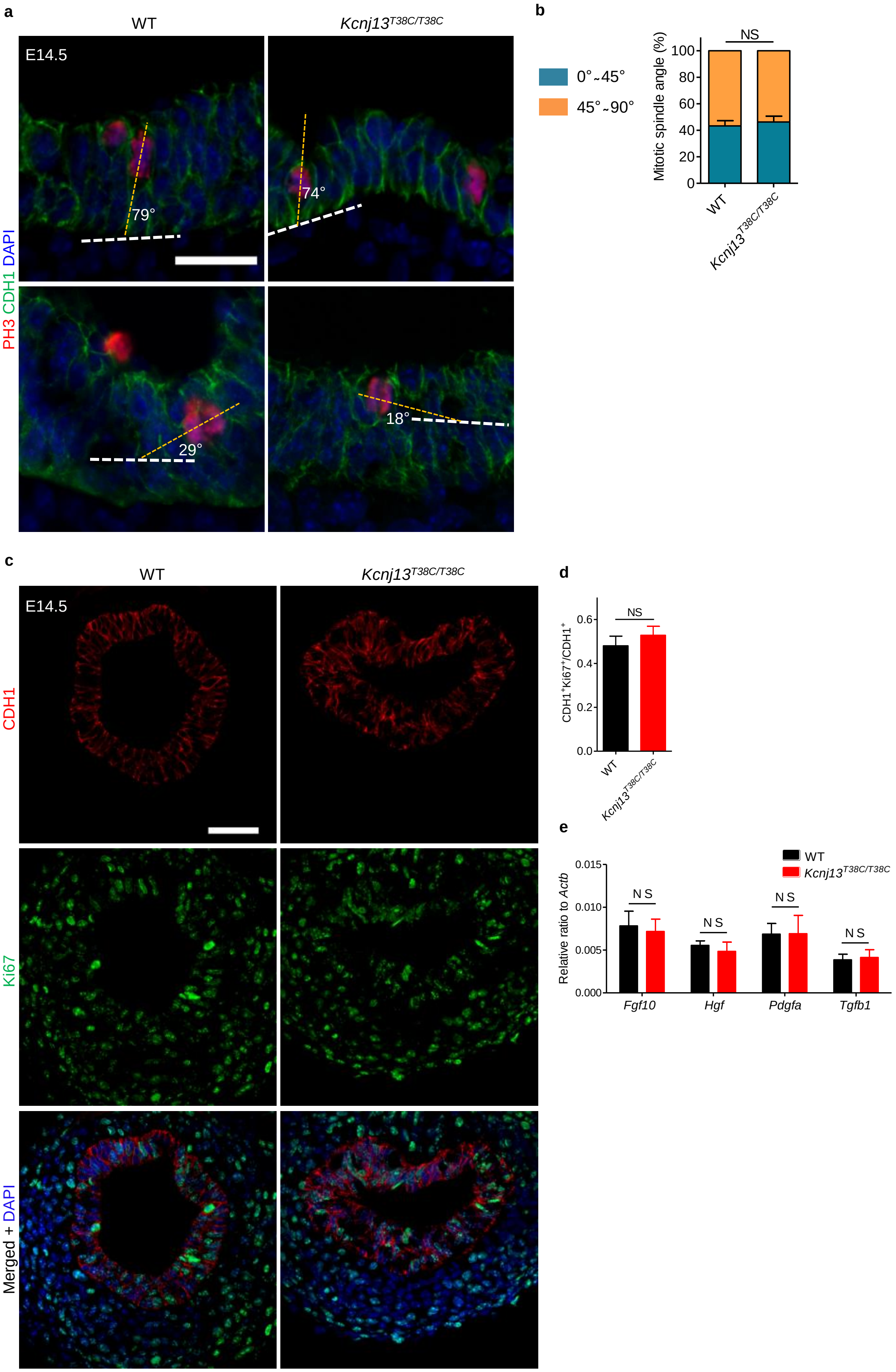

Supplementary Figure 9. *Kcnj13*$^{T38C/T38C}$ mice exhibit WT-like mitotic spindle orientation and proliferation of tracheal epithelial cells. (**a**) Immunostaining for PH3 (red) and CDH1 (green) and

DAPI staining (blue) of transverse sections of E14.5 WT (n=7) and *Kcnj13*$^{T38C/T38C}$ (n=7) tracheas. (**b**) Quantification of the mitotic spindle angle of E14.5 WT (n=7) and *Kcnj13*$^{T38C/T38C}$ (n=7) dividing epithelial cells. 30 cells were analyzed per animal. (**c**) Immunostaining for CDH1 (red) and Ki67 (green) and DAPI staining (blue) of transverse sections of E14.5 WT (n=7) and *Kcnj13*$^{T38C/T38C}$ (n=7) tracheas. (**d**) Percentage of CDH1^{+} cells which are Ki67^{+}. (**e**) RT–qPCR analysis of *Fgf10, Hgf, Pdgfa* and *Tgfb1* expression in E14.5 WT (n=6) and *Kcnj13*$^{T38C/T38C}$ (*n*=6) tracheas. Scale bars: 50 μm (**c**), 20 μm (**a**). NS, not significant; Unpaired Student's *t*-test, mean ± s.d.

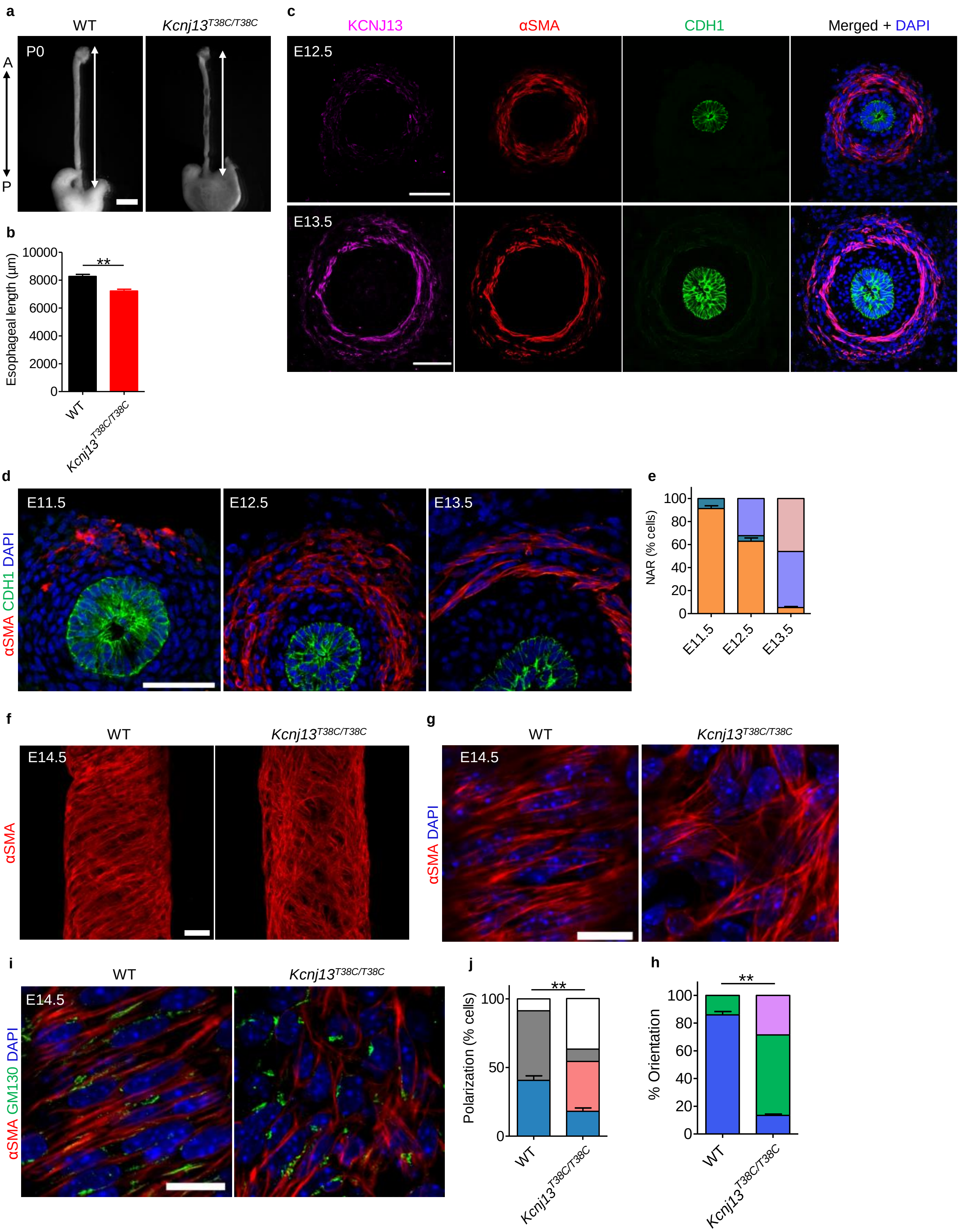

Supplementary Figure 10. ***Kcnj13$^{T38C/T38C}$*** **mice exhibits esophageal elongation defects with altered SM cell alignment and polarity.** (**a**) Representative images of P0 WT (n=17) and *Kcnj13$^{T38C/T38C}$*

(n=17) esophagi. Double-sided arrows indicate esophageal length. (**b**) Quantification of P0 WT (n=17) and *Kcnj13*$^{T38C/T38C}$ (n=17) esophageal length. (**c**) Immunostaining for KCNJ13 (violet), αSMA (red) and CDH1 (green) and DAPI staining (blue) of transverse sections of E12.5 (n=5) and E13.5 (n=5) WT esophagi. (**d**) Immunostaining for αSMA (red) and CDH1 (green) and DAPI staining (blue) of transverse sections of WT esophagi from E11.5 (n=6) to E13.5 (n=5). (**e**) Quantification of WT esophageal SM cell NAR from E11.5 (n=6) to E13.5 (n=5). (**f**) Immunostaining for αSMA (red) in E14.5 WT (n=8) and *Kcnj13*$^{T38C/T38C}$ (n=8) esophagi. (**g**) Immunostaining for αSMA (red) and DAPI staining (blue) in E14.5 WT (n=8) and *Kcnj13*$^{T38C/T38C}$ (n=8) esophagi. (**h**) Quantification of E14.5 WT (n=8) and *Kcnj13*$^{T38C/T38C}$ (n=8) esophageal SM cell orientation. (**i**) Immunostaining for αSMA (red) and GM130 (green) and DAPI staining (blue) in E14.5 WT (n=8) and *Kcnj13*$^{T38C/T38C}$ (n=8) esophagi. (**j**) Quantification of E14.5 WT (n=8) and *Kcnj13*$^{T38C/T38C}$ (n=8) Golgi-apparatus position relative to the nucleus. Scale bars: 1000 µm (**a**), 50 µm (**c, d, f**), 20 µm (**g, i**). $^{**}P < 0.01$; Unpaired Student's *t*-test, mean ± s.d. A, Anterior; P, Posterior.

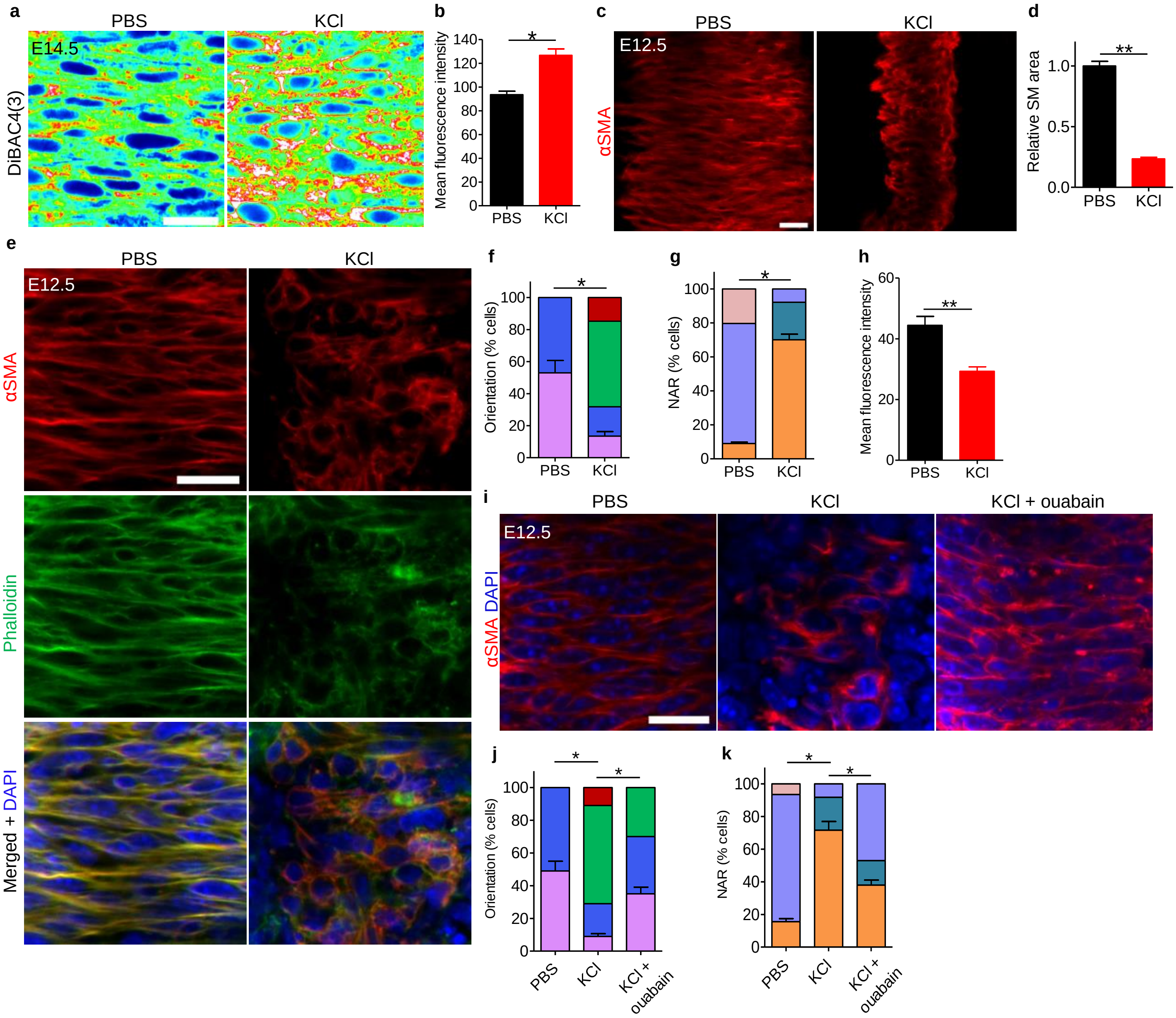

Supplementary Figure 11. Elevated extracellular potassium concentration in tracheas leads to SM cell phenotypes similar to those observed in *Kcnj13*$^{T38C/T38C}$ mice. **(a)** DiBAC4(3) fluorescence in dorsal views of E14.5 tracheal SM cells after PBS (n=9) or 40 mM KCl (n=9) treatment. (**b**) Quantification of mean DiBAC4(3) fluorescence intensity in E14.5 tracheal SM cells after PBS (n=9) or 40 mM KCl (n=9) treatment. (**c**) Immunostaining for αSMA (red) in dorsal views of E12.5 tracheas after a 48 hour PBS (n=9) or 40 mM KCl (n=9) treatment. (**d**) Quantification of SM area in E12.5 tracheas after a 48 hour PBS (n=9) or 40 mM KCl (n=9) treatment. (**e**) Immunostaining for αSMA (red) and phalloidin (green) and DAPI (blue) staining in dorsal views of E12.5 tracheas after a 48 hour PBS (n=9) or 40 mM KCl (n=9) treatment. (**f**) Quantification of E12.5 tracheal SM cell orientation after a 48 hour PBS (n=9) or 40 mM KCl (n=9) treatment. (**g**) Quantification of E12.5 tracheal SM cell NAR after a 48 hour PBS (n=9) or 40 mM KCl (n=9) treatment. (**h**) Quantification of mean phalloidin fluorescence intensity in E12.5 tracheal SM cells after a 48 hour PBS (n=9) or 40 mM KCl (n=9) treatment. (**i**) Immunostaining for αSMA (red) and DAPI staining (blue) in dorsal views of E12.5 WT tracheas after a 48 hour PBS (n=24), 40 mM KCl (n=12) or 40 mM KCl plus 200 µM ouabain (pre-incubated for 30 min) (n=12) treatment. (**j**) Quantification of SM cell orientation of E12.5 WT tracheas after a 48 hour PBS (n=12), 40 mM KCl (n=12) or 40 mM KCl plus 200 µM ouabain (pre-incubated for 30 min) (n=12) treatment. (**k**) Quantification of SM cell NAR of E12.5 WT tracheas after a 48 hour PBS (n=12), 40 mM KCl (n=12) or 40 mM KCl plus 200 µM ouabain (pre-incubated for 30 min) (n=12) treatment. Scale bars: 20 µm (**a, c, e, i**). *$P < 0.05$; **$P < 0.01$; Unpaired Student's *t*-test, mean ± s.d.

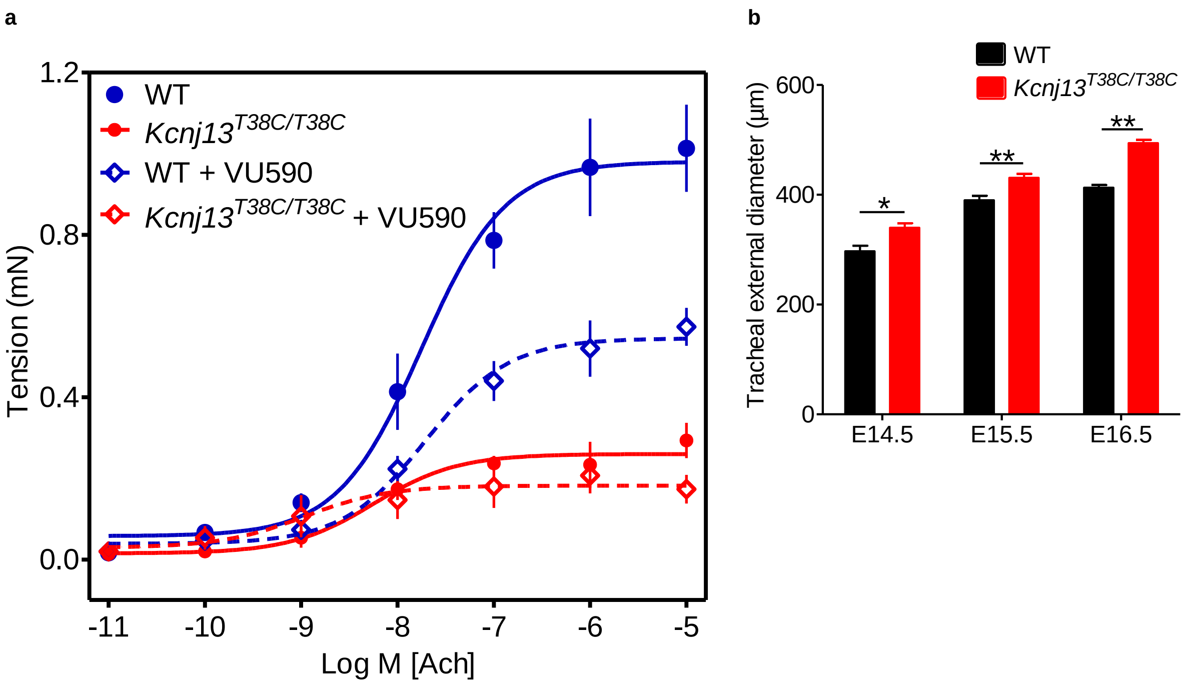

Supplementary Figure 12. ***Kcnj13**$^{T38C/T38C}$* **mice exhibit compromised tracheal contraction and expanded tube diameter.** (**a**) Tracheal contractile force measurements of P0 WT (n=12), *Kcnj13*$^{T38C/T38C}$ (n=12), as well as WT (n=12) and *Kcnj13*$^{T38C/T38C}$ (n=12) after a 10 min 50 µM VU590 treatment. (**b**) Quantification of WT (n=8) and *Kcnj13*$^{T38C/T38C}$ (n=8) external diameter of the anterior trachea at several embryonic stages. *$P < 0.05$; **$P < 0.01$; Unpaired Student's *t*-test, mean ± s.d.

Uncropped images related to Fig. 6c

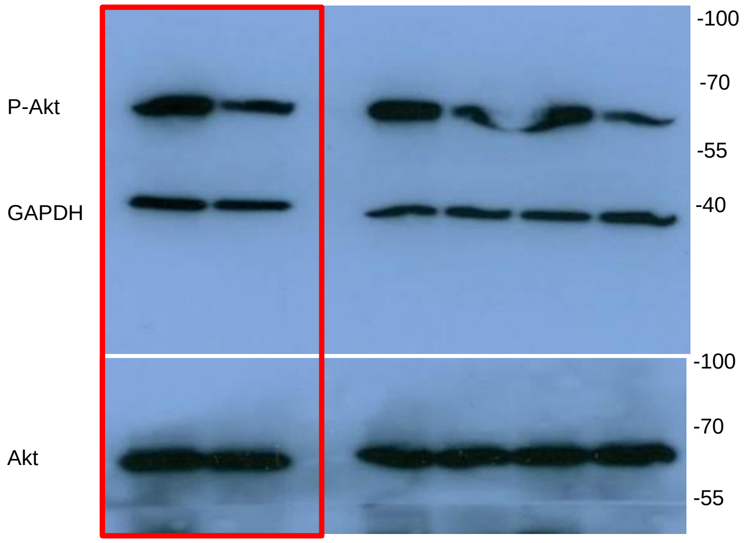

Uncropped images related to Fig. 6e

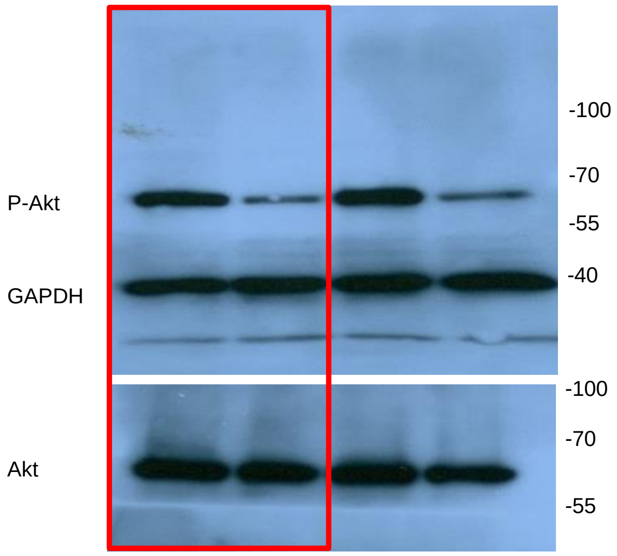

Supplementary Figure 13. Uncropped images related to western blotting data.

Supplementary Tables

Sample Name	Assay Name	Ct mean
E11.5	*Kcnj13*	31.79
E11.5	*Actb*	24.52
E12.5	*Kcnj13*	31.71
E12.5	*Actb*	24.46
E13.5	*Kcnj13*	32.29
E13.5	*Actb*	25.03

Supplementary Table 1. RNA expression levels were analyzed by RT-qPCR; Ct values listed for Supplementary Fig. 4a.

Sample Name	Assay Name	Ct mean
E14.5 WT	*Sox9*	26.31
E14.5 *Kcnj13*$^{T38C/T38C}$	*Sox9*	26.70
E14.5 WT	*Actb*	21.60
E14.5 *Kcnj13*$^{T38C/T38C}$	*Actb*	21.89

Supplementary Table 2. RNA expression levels were analyzed by RT-qPCR; Ct values listed for Supplementary Fig. 5f.

Sample Name	Assay Name	Ct mean
E14.5 WT	*Fgf10*	30.75
E14.5 *Kcnj13*$^{T38C/T38C}$	*Fgf10*	30.93
E14.5 WT	*Hgf*	31.22
E14.5 *Kcnj13*$^{T38C/T38C}$	*Hgf*	31.51
E14.5 WT	*Pdgfa*	30.93
E14.5 *Kcnj13*$^{T38C/T38C}$	*Pdgfa*	31.02
E14.5 WT	*Tgfb1*	30.56
E14.5 *Kcnj13*$^{T38C/T38C}$	*Tgfb1*	30.60
E14.5 WT	*Actb*	23.72
E14.5 *Kcnj13*$^{T38C/T38C}$	*Actb*	23.78

Supplementary Table 4. RNA expression levels were analyzed by RT-qPCR; Ct values listed for Supplementary Fig. 9e.

Sample Name	Assay Name	Ct mean
E14.5 WT	*Wnt5a*	27.67
E14.5 *Kcnj13*$^{T38C/T38C}$	*Wnt5a*	28.19
E14.5 WT	*Ror2*	29.92
E14.5 *Kcnj13*$^{T38C/T38C}$	*Ror2*	30.70
E14.5 WT	*Fzd4*	28.20
E14.5 *Kcnj13*$^{T38C/T38C}$	*Fzd4*	28.57
E14.5 WT	*Fzd6*	31.08
E14.5 *Kcnj13*$^{T38C/T38C}$	*Fzd6*	31.13
E14.5 WT	*Fzd8*	28.29
E14.5 *Kcnj13*$^{T38C/T38C}$	*Fzd8*	28.88
E14.5 WT	*Vangl1*	29.22
E14.5 *Kcnj13*$^{T38C/T38C}$	*Vangl1*	29.86
E14.5 WT	*Dvl1*	31.88
E14.5 *Kcnj13*$^{T38C/T38C}$	*Dvl1*	32.14
E14.5 WT	*Dvl2*	29.86
E14.5 *Kcnj13*$^{T38C/T38C}$	*Dvl2*	30.59
E14.5 WT	*Dvl3*	29.67
E14.5 *Kcnj13*$^{T38C/T38C}$	*Dvl3*	30.14
E14.5 WT	*Celsr1*	31.99
E14.5 *Kcnj13*$^{T38C/T38C}$	*Celsr1*	32.44
E14.5 WT	*Celsr2*	30.77
E14.5 *Kcnj13*$^{T38C/T38C}$	*Celsr2*	30.90
E14.5 WT	*Pk1*	28.66
E14.5 *Kcnj13*$^{T38C/T38C}$	*Pk1*	29.22
E14.5 WT	*Actb*	23.21
E14.5 *Kcnj13*$^{T38C/T38C}$	*Actb*	23.77

Supplementary Table 3. RNA expression levels were analyzed by RT-qPCR; Ct values listed for Supplementary Fig. 6.
