# Supplementary material for: The potassium channel KCNJ13 is essential for smooth muscle cytoskeletal organization during mouse tracheal tubulogenesis
Source: Nat Commun. 2018 Jul 19;9:2815. doi: 10.1038/s41467-018-05043-5 (PMC6052067; doi:10.1038/s41467-018-05043-5)
Supplement: Supplementary file 2 — Description of Additional Supplementary Files [file 41467_2018_5043_MOESM2_ESM.docx]

**Description of Additional Supplementary Files**

File Name: Supplementary Movie 1

Description: Neonatal *Kcnj13^T38C/T38C^* mice (n=15) exhibit respiratory distress
